# Overexpression of α-Synuclein by Oligodendrocytes in Transgenic Mice Does Not Recapitulate the Fibrillar Aggregation Seen in Multiple System Atrophy

**DOI:** 10.3390/cells9112371

**Published:** 2020-10-29

**Authors:** Florent Laferrière, Xin He, Federica Zinghirino, Evelyne Doudnikoff, Emilie Faggiani, Wassilios G. Meissner, Erwan Bezard, Francesca De Giorgi, François Ichas

**Affiliations:** 1 CNRS, Institut des Maladies Neurodégénératives, UMR 5293, 33076 Bordeaux, France; florent.laferriere@u-bordeaux.fr (F.L.); xin.he@u-bordeaux.fr (X.H.); federica.zinghirino@phd.unict.it (F.Z.); evelyne.doudnikoff@u-bordeaux.fr (E.D.); emilie.faggiani@u-bordeaux.fr (E.F.); wassilios.meissner@chu-bordeaux.fr (W.G.M.); erwan.bezard@u-bordeaux.fr (E.B.); 2 Institut des Maladies Neurodégénératives, UMR 5293, Université de Bordeaux, 33076 Bordeaux, France; 3 Department of Neurology, Sheng Jing Hospital of China Medical University, Shenyang 110004, China; 4 Dipartimento di Scienze Biomediche e Biotecnologiche, BIOMETEC, Università degli Studi di Catania, 95123 Catania, Italy; 5 Service de Neurologie, CRMR Atrophie Multisystématisée, CHU Bordeaux, 33000 Bordeaux, France; 6 INSERM, Laboratoire de Neurosciences Expérimentales et Cliniques, U-1084, Université de Poitiers, 86000 Poitiers, France

**Keywords:** α-synuclein, multiple system atrophy, GCIs

## Abstract

The synucleinopathy underlying multiple system atrophy (MSA) is characterized by the presence of abundant amyloid inclusions containing fibrillar α-synuclein (α-syn) aggregates in the brains of the patients and is associated with an extensive neurodegeneration. In contrast to Parkinson’s disease (PD) where the pathological α-syn aggregates are almost exclusively neuronal, the α-syn inclusions in MSA are principally observed in oligodendrocytes (OLs) where they form glial cytoplasmic inclusions (GCIs). This is intriguing because differentiated OLs express low levels of α-syn, yet pathogenic amyloid α-syn seeds require significant amounts of α-syn monomers to feed their fibrillar growth and to eventually cause the buildup of cytopathological inclusions. One of the transgenic mouse models of this disease is based on the targeted overexpression of human α-syn in OLs using the PLP promoter. In these mice, the histopathological images showing a rapid emergence of S129-phosphorylated α-syn inside OLs are considered as equivalent to GCIs. Instead, we report here that they correspond to the accumulation of phosphorylated α-syn monomers/oligomers and not to the appearance of the distinctive fibrillar α-syn aggregates that are present in the brains of MSA or PD patients. In spite of a propensity to co-sediment with myelin sheath contaminants, the phosphorylated forms found in the brains of the transgenic animals are soluble (>80%). In clear contrast, the phosphorylated species present in the brains of MSA and PD patients are insoluble fibrils (>95%). Using primary cultures of OLs from PLP-αSyn mice we observed a variable association of S129-phosphorylated α-syn with the cytoplasmic compartment, the nucleus and with membrane domains suggesting that OLs functionally accommodate the phospho-α-syn deriving from experimental overexpression. Yet and while not taking place spontaneously, fibrillization can be seeded in these primary cultures by challenging the OLs with α-syn preformed fibrils (PFFs). This indicates that a targeted overexpression of α-syn does not model GCIs in mice but that it can provide a basis for seeding aggregation using PFFs. This approach could help establishing a link between α-syn aggregation and the development of a clinical phenotype in these transgenic animals.

## 1. Introduction

Multiple system atrophy (MSA) is a fatal neurodegenerative disorder, characterized by a synucleinopathy that consists in the abundant presence of fibrillar α-synuclein (α-syn) in oligodendrocytes (OLs) forming glial cytoplasmic inclusions (GCIs) and to a lesser extent in neurons [1,2,3,4]. Based on these observations and similar to other synucleinopathies like Parkinson’s disease (PD), α-syn self-assembly as amyloid fibrils is thought to represent a key pathologic event capable to account for the autocatalytic spread of the disease and thus represents a prominent candidate target for disease modification in MSA.

The origin of α-syn in OLs remains the matter of an ongoing controversy [5]. During differentiation the OL progenitors progressively turn down α-syn expression [6]. In addition and even if the presence of α-syn mRNA has been reported in these cells, its levels are not statistically different in OLs from healthy vs. MSA brains [7]. Thus, the contribution of post-translational mechanisms has been proposed to account for the accumulation and the aggregation of α-syn in OLs, among which: the relocation of the p25α protein from the myelin sheath to the cytoplasm secondarily trapping α-syn [8,9] and alterations in the exosomal export of α-syn causing its intracellular accumulation [10]. An alternative possibility is that in MSA α-syn could be imported by OLs by virtue of a cell-to-cell transfer of α-syn from neurons that strongly express α-syn [11,12] and show abundant α-syn oligomers in MSA patient brains [13].

Although it is unclear which is the origin of the pathological accumulation and aggregation of α-syn in the OLs, most of the experimental animal models for MSA are based on a targeted overexpression of human α-syn in these cells [14,15,16]. In these transgenic models the question of why and how α-syn tends to accumulate in OLs is circumvented and only the events second to the experimental overexpression of α-syn in OLs can be analyzed.

In spite of this shortcut, the attractiveness of these models lies in their capability to recapitulate certain key clinical and neurodegenerative features distinctive of MSA. They have thus been adopted to investigate the pathogenesis and pathophysiology of MSA, to identify new treatment targets and to validate the most promising compounds before clinical testing [5,17,18,19,20,21]. In addition, they also possibly offer an in vivo context to address the question of the causal link existing between the fibrillization of α-syn, the development of neuropathological inclusions (GCIs) and the observed clinical phenotype. This causal relationship still remains unclear in MSA as well as in other synucleinopathies.

One of these transgenic mouse models uses the proteolipid protein promoter (PLP) to drive α-syn expression in mice bred on a C57/BL6 background (PLP-αSyn) [14]. The brains of these mice exhibit a generalized OL α-syn burden with extensive S129 phosphorylation [14]. Since this post-translational modification is associated with GCIs in MSA [22,23], it is thus widely accepted that this phosphorylated α-syn burden indicates the emergence of “experimental GCIs” in this animal model [24]. This is seemingly in line with the observation that MSA-like clinical features (motor impairment, autonomic dysfunction) and neurodegeneration (i.e., DA neuron loss) develop in PLP-αSyn mice [25,26,27,28,29].

We thus put under closer scrutiny the α-syn pools found in the OLs of these transgenic animals and compared these pools with the α-syn extracted from the brains of control subjects, MSA and PD patients, as well as with synthetic preformed fibrils (PFFs) made of recombinant human α-syn.

## 2. Materials and Methods

### 2.1. Animals

Mice expressing human α-synuclein in oligodendrocytes under the control of the proteolipid promoter (PLP-αSyn) were previously generated on a C57BL/6J background [14] and kindly provided by Dr. P.O. Fernagut (University of Poitiers, Poitiers, France). Both PLP-αSyn and C57/BL6J mice were bred and maintained in the IMN animal facilities. All mice were housed in a temperature-controlled (22 °C) and light-controlled environment on a 12-h light/12-h dark cycle with access to food and water ad libitum. Animal sacrifice was conducted in accordance with the European Communities Council Directive (2010/63/EU) for care of laboratory animals

### 2.2. Primary Culture of Cortical Neurons and High Content Analysis

The list of chemicals appears in Supplementary Table 1. Timed pregnant mice were used. Brain cortices were harvested from E18 mouse embryos and dissociated enzymatically and mechanically (using the neuronal tissue dissociation kit, C-Tubes and an Octodissociator with heaters, Miltenyi Biotech, Bergisch Gladbach, Germany) to yield a homogenous cell suspension. The cells were then plated at 20,000 per well in 96-well plates (Corning, Biocoat Poly-D lysine Imaging plates, Corning, New York, NY, USA) in neuronal medium (Neuronal Macs medium, Miltenyi Biotech, Bergisch Gladbach, Germany) containing 0.5% Penicillin/Streptomycin, 0.5 mM alanyl-glutamine and 2% Neurobrew supplement (Miltenyi Biotech, Bergisch Gladbach, Germany). The medium was changed by 1/3 every 3 days, until a maximum of 30 days in vitro (DIV). When mentioned, isolated oligodendrocyte precursor cells (OPCs) (5000 per well) were added at DIV 2. For the induction of the experimental synucleinopathy, 10 nM (equivalent monomeric concentration) of α-syn PFFs were added at DIV 7 [30]. PFFs were templated on the 1B polymorph [30] (PFFs and PFFs#1) or on the iso3 polymorph (PFF#2) [30] by seeding the monomers with 1% *w/w* template prior to the fibrillization as described in Reference [30]. High Content Analysis was performed on multichannel fluorescence images acquired 20× using the generic analysis module of the Incucyte S3 and Top-Hat cellular segmentation was based on the fluorescence signal corresponding to the antibody of interest. For the quantification of the neuronal synucleinopathy we used the neurite segmentation module and based the recognition process on the fluorescence corresponding to the EP1536Y signal. This segmentation methodology is equivalent to the sequential application of two filters: one fluorescence-intensity-based that retains the EP1536Y-positive pixels above a certain threshold and one morphological that only retains the pixels groups forming linear objects (neurites). Settings were determined to allow the specific detection of the neuronal neuritic synucleinopathy and filter out the EP1536Y signal present elsewhere. For the sake of simplicity, we thus call it neuronal synucleinopathy in Figure 6. The parameters were as follows: Color Neurites panel (EP1536Y); cell-body cluster segmentation: Top Hat, Radius 20 µm, Threshold RCU 2; Cleanup: Min Cell Width 7 (other parameters 0); Cell-body cluster filter: none; Neurite Parameters: coarse sensitivity 10, fine sensitivity 0.5, Width 1 µm.

### 2.3. Primary Culture of Mouse Oligodendrocytes

Timed pregnant mice were used. Brain cortices were harvested from E18 mouse embryos and dissociated enzymatically and mechanically (using Neuronal tissue dissociation kit, C-Tubes and Octodissociator with heaters, all from Miltenyi Biotech, Bergisch Gladbach, Germany) to yield a homogenous cell suspension.

Oligodendrocyte precursor cells were then isolated from this suspension based on the expression of CD140a using magnetic separation (CD140a (PDGFRα) MicroBead Kit, MACS™ Columns and MACS™ Separators, all from Miltenyi Biotech, Bergisch Gladbach, Germany) using the protocol made available by Miltenyi Biotech.

The cells were then plated at 5000 per well in 96-well Biocoat plates (poly L-lysine, Corning, New York, NY, USA) in neuronal medium (Neuronal Macs medium, Miltenyi Biotech, Bergisch Gladbach, Germany) containing 0.5% penicillin-streptomycin, 0.5 mM alanyl-glutamine and 2% Neurobrew supplement (Miltenyi Biotech, Bergisch Gladbach, Germany), FGF 20 ng/mL, PDGF-A 20 ng/mL (Miltenyi Biotech, Bergisch Gladbach, Germany). Medium was changed every 3 days by one third. From DIV 7 on, medium was progressively replaced by Brainphys (Stemcell Technologies, Vancouver, BC, Canada) supplemented with Neurobrew 2%, FGF 20 ng/mL, PDGF-A 20 ng/mL. At DIV 26 PDGF-A was omitted and replaced with CTNF 10 ng/mL (Miltenyi Biotech, Bergisch Gladbach, Germany) for differentiation.

### 2.4. Recombinant α-Syn Expression and Purification

E. coli strain BL21 (DE3) plysS was transformed with pET24-α-Syn vector by electroporation and plated onto LB agar plate containing 30 µg/mL Kanamycin. A pre-culture in 5 mL LB medium was inoculated with one clone and incubated at 37 °C under 200 rpm shaking for 4 h. The expression on α-syn was carried out in M9 minimal medium containing 2 g/L of 13C Glucose and 1 g/L of NH_4_Cl as carbon and nitrogen sources. Cells from LB pre-culture were recovered by centrifugation (1000× *g*–10 min) and used for inoculating 200 mL of M9 medium. Cells were grown overnight at 37 °C under 200 rpm shaking and then diluted in 2 L of culture. Protein expression was induced by adding 1 mM IPTG during exponential phase, evaluated by Optical Density at 600 nm reaching 0.8. Cells were harvested after 4–5 h of culture at 37 °C by 6000× *g* centrifugation (JLA 8.1 Beckman Coulter) and pellet was kept at −20 °C before purification.

Pellet was thawed in 10 mM Tris-HCl (pH 8.0), 1 mM EDTA and 1 mM PMSF, Pierce Complete EDTA-free protease inhibitors tablet (Thermofisher) buffer and sonicated 3 times for 45 sec (Bandelin Sonoplus–VS70T probe) prior to be centrifuging. The supernatant was boiled for 20 min and centrifuged. Streptomycin sulphate was added to supernatant to a final concentration of 10 mg/mL and the solution was stirred for 15 min at 4 °C and then centrifuged. Ammonium sulphate was added to supernatant to a final concentration of 360 mg/mL and the mixture was stirred for 15 min at 4 °C before being centrifuged. These four centrifugations were performed at 20,000 rpm for 30 min and at 4 °C with Beckman Coulter JA-25.5 rotor. The pellet was resuspended in 25 mM Tris-HCl (pH 7.70) and dialyzed against the same buffer to eliminate salts. The dialyzed sample was injected onto HiTrap Q HP column previously equilibrated with 25 mM Tris-HCl (pH 7.70) and α-synuclein was eluted around 250 µM of NaCl by steps from 0 mM to 500 mM NaCl with AKTA pure system. Fractions containing the protein were dialyzed against 20 mM Tris-HCl (pH 7.40) and 100 mM NaCl buffer before to be loading onto HiLoad 26/600 Superdex 75 pg column equilibrated with the same buffer with AKTApur system. Monomeric fractions were collected and concentrated if needed by using Vivaspin 15R 2 kDa cut off concentrator (Sartorius Stedim, Gottingen, Germany). Purification fractions were checked by using Poly Acrylamide Gel Electrophoresis Tris-tricine 13% dying with ProBlueSafe Strain. Protein concentration was evaluated spectrophotometrically by using absorbance at 280 nm and extinction coefficient of 5960 M^−1^.cm^−1^.

### 2.5. α-syn Fibrillization

Solutions of monomeric α-syn at 4–5 mg/mL were sterilized by filtration with 0.22 µm Millipore single use filters and stored in sterile 15 mL conical falcon tubes at 4 °C. Sterilized stock was then distributed into safe-lock “Biopur” individually sterile-packaged 1.5 mL Eppendorf tubes as 500 µL aliquots. The tubes were cap-locked and additionally sealed with parafilm. All the previous steps were performed aseptically in a particle-free environment, under a microbiological safety laminar flow hood. For comparative fibrillizations, all the samples were loaded simultaneously in a Thermomixer (Eppendorf, Hamburg, Germany) in a 24 position 1.5 mL Eppendorf tube holder equipped with a heating lid. Temperature was set to 37 °C and shaking to 2000 rpm. Sampling for measurements during the fibrillization process was done by temporarily returning the samples under the microbiological safety laminar flow hood.

Samples from the fibrillized α-syn aliquots were diluted to 0.1 mg/mL in 100 µL in PBS and distributed in cap-locked sterile 0.5 mL PCR tubes (Thermo Fisher Scientific, Waltham, MA, USA). Sonication was performed at 25 °C in a BioruptorPlus water bath sonicator (Diagenode, Seraing, Belgium) equipped with thermostatic control and automated tube carousel rotator. The sonication power was set to “high” and 10 cycles of 30 s “on” followed by 10 s “off” were applied.

### 2.6. Immunofluorescence

Cells were fixed with 4% (*w*/*v*) paraformaldehyde/4% (*w*/*v*) sucrose for 15 min, permeabilized and blocked with 3% (*w*/*v*) BSA/0.1% (vol/vol) TX-100 for 15 min and incubated at 4 °C overnight with primary antibody diluted in the blocking buffer (Table 1). Secondary antibody was incubated for 1h at 37 °C (Table 1).

### 2.7. Brain Sections Staining

The brains were perfused with saline, post-fixed for 3 days in 10 mL of 4% paraformaldehyde at 4 °C, cryoprotected in gradient 20% sucrose in PBS before being frozen by immersion in a cold isopentane bath (−60 °C) for at least 5 min and stored immediately at −80 °C until sectioning for immunofluorescence analysis. After serial sectioning, the sections were stained using the primary antibodies EP1536Y (Abcam) for detecting phospho-S129 positive α-syn and MJFR1 for human α-syn. The slides were acquired using Incucyte S3 High Content Imager (Sartorius, Göttingen, Germany) with a home-made 3D-printed slide holder for IF.

### 2.8. SarkoSpin Fractionation

SarkoSpin procedure was slightly adapted from previously published protocols [31,32]. The samples were obtained from brains collected in a Brain Donation Program of the Brain Bank “GIE NeuroCEB” (Neuro-CEB BB-0033-00011). The consents were signed by the patients themselves or their next of kin in their name, in accordance with the French Bioethical Laws. The Brain Bank GIE NeuroCEB has been declared at the Ministry of Higher Education and Research and has received approval to distribute samples (agreement AC-2013-1887). Human cortices (cingulate gyrus) were dissected from freshly frozen post-mortem brain samples from *n* = 3 control, sporadic PD or MSA subjects respectively. For mouse brain samples, at the age indicated, mice were anesthetized and intracardially perfused with 0.9% saline. Brains were quickly removed and homogenized. Mouse and human brain tissues were homogenized at 10% (*w*/*v*) in solubilization buffer (SB): 10 mM Tris pH 7.5, 150 mM NaCl, 0.1 mM EDTA, 1 mM DTT, Complete EDTA-free protease inhibitors (Roche, Basel, Switzerland) and PhosSTOP phosphatase inhibitors (Roche) using a gentleMACS Octo Dissociator (Miltenyi Biotec, Bergisch Gladbach, Germany) with M Tubes and the Protein extraction program. Stock preparations of α-syn amyloid fibrils (5 mg/mL in TBS) were diluted with SB buffer to obtain 1 µg in each centrifugation sample in an equivalent volume to brain homogenates. Samples were mixed 1:1 with SB 4% or 2% (*w*/*v*) N-lauroyl-sarcosine (sarkosyl, Sigma-Aldrich, Saint-Louis, MO, USA) for human brain or mouse brain and PFF respectively, 2 U.μL^–1^ Benzonase (Novagen, Burlington, MA, USA) and 4 mM MgCl_2_, reaching a final volume of 500 μL. SarkoSpin solubilization was then performed by incubating the samples at 37 °C under constant shaking at 600 rpm (Thermomixer, Eppendorf, Hamburg, Germany) for 45 min. Solubilized samples were then mixed 1:1 with SB 40% (*w*/*v*) sucrose, without sarkosyl MgCl_2_ and Benzonase, in 1 mL polycarbonate ultracentrifuge tubes (Beckman, Brea, CA, USA) and centrifuged at 250,000× *g* for 1 h at room temperature with a TLA 120.2 rotor using an Optima XP benchtop ultracentrifuge (Beckman, Brea, CA, USA). Supernatant were collected by pipetting. Pellets were resuspended directly in the tube with 100 µL of the buffer corresponding to the supernatant (SB 0.5 or 1% sarkosyl and 0 or 20% sucrose) and mixed with the same buffer in a fresh tube for reaching 1ml (equal volumes to supernatant).

### 2.9. Sedimentation Velocity Gradient Fractionation

Sedimentation velocity gradient fractionations were performed as published previously [31,32,33]. Briefly, a volume of 400 µL of SarkoSpin solubilized samples was loaded on top of a 11 mL continuous 5–20% iodixanol gradient (Optiprep, 60% (*w*/*v*) iodixanol, Sigma-Aldrich, Saint-Louis, MO, USA) in SB buffer with 0.5% (*w*/*v*) sarkosyl linearized directly in ultracentrifuge 12 mL tubes (Seton scientific, Petaluma, CA, USA) with a Gradient Master (Biocomp instruments, Fredericton, NB, Canada). The gradients were centrifuged at 200,000× *g* for 2.5 h at room temperature in a swinging-bucket SW-41 Ti rotor using an Optima LE-80K ultracentrifuge (Beckman, Brea, CA, USA). Gradients were then segregated into 16 equal fractions from the top using a piston fractionator (Biocomp instruments, Fredericton, NB, Canada) and a fraction collector (Gilson, Middleton, WI, USA). Fractions were aliquoted for further analysis of their content by immunoblot. Gradient linearity was verified by refractometry.

### 2.10. Analysis of the Protein Contents of Sarkospin and Velocity Fractions by Filter Trap

For filter trap assays, native fractions were spotted onto nitrocellulose 0.2 µm membranes (Protran, GE) using a dot blot vacuum device (Whatman, Maidstone, UK). Nitrocellulose membranes were fixed for 30 min in PBS with PFA 0.4% (*v/v*) (Sigma-Aldrich, Saint-Louis, MO, USA) final concentration. After three washes with PBS, membranes were blocked with 5% (*w*/*v*) skimmed powder milk in PBS-Tween 0.5% (*v/v*) and probed with primary and secondary antibodies in PBS-Tween with 4% (*w*/*v*) BSA (Table 1). Immunoreactivity was whether visualized by chemiluminescence or infrared using Clarity ECL and Chemidoc (Biorad, Hercules, CA, USA) or Odissey systems (Li-Cor, Lincoln, NE, USA)) respectively.

### 2.11. Crosslinking and Western Blot

Pooled (*n* = 3) WT or PLP-αSyn mouse brain homogenates were prepared as described above. Samples of equal protein concentration (determined with BCA) were denucleated by mild centrifugation at 800× *g* for 5 min at 4 °C. Supernatants were treated 15 min at room temperature 1 U.μL^–1^ Benzonase (Novagen, Burlington, MA, USA) with 2 mM MgCl_2_ final concentrations. When specified, samples were solubilized 30 min on ice with SB 0.25% (*v/v*) Triton-X final concentration, equal volumes of SB without detergent was added to non-solubilized samples incubated under the same conditions. When specified, samples were crosslinked with 2 mM final concentration disuccinimidyl glutarate (DSG, Sigma) at room temperature for 15 min before stopping the reaction with 100 mM final concentration of Tris. Non-crosslinked samples were treated in the same conditions without DSG. Aliquots of the resulting samples were added Laemmli 1X prior to denaturation at 95 °C for 5 min and loaded on Mini-Protean TGX 12% gels (Biorad) followed by SDS-PAGE electrophoresis. Gels were transferred on nitrocellulose 0.2 µm membranes with Trans-Blot Turbo transfer system (Biorad) using the Mixed molecular weight program. Membranes were fixed with PFA and proteins were immunolabelled with infrared secondary antibodies as described for filter trap. After detection with Odyssey system (Li-Cor), whole lane signal intensity profiles were quantified and plotted on graphs with MetaMorph software (Molecular Devices, San José, CA, USA).

### 2.12. Antibodies

All the antibodies used and shown in the study are summarized in Table 1. All the plates were acquired and analyzed using an Incucyte S3 High Content Imager (Sartorius, Göttingen, Germany).

## 3. Results and Discussion

### 3.1. S129-phosphorylated α-syn Species Found in the Brain of PLP-αSyn Mice Are Distinct from the Amyloid forms Extracted from PD and MSA Brains and from Recombinant PFFs

**Figure 1:**
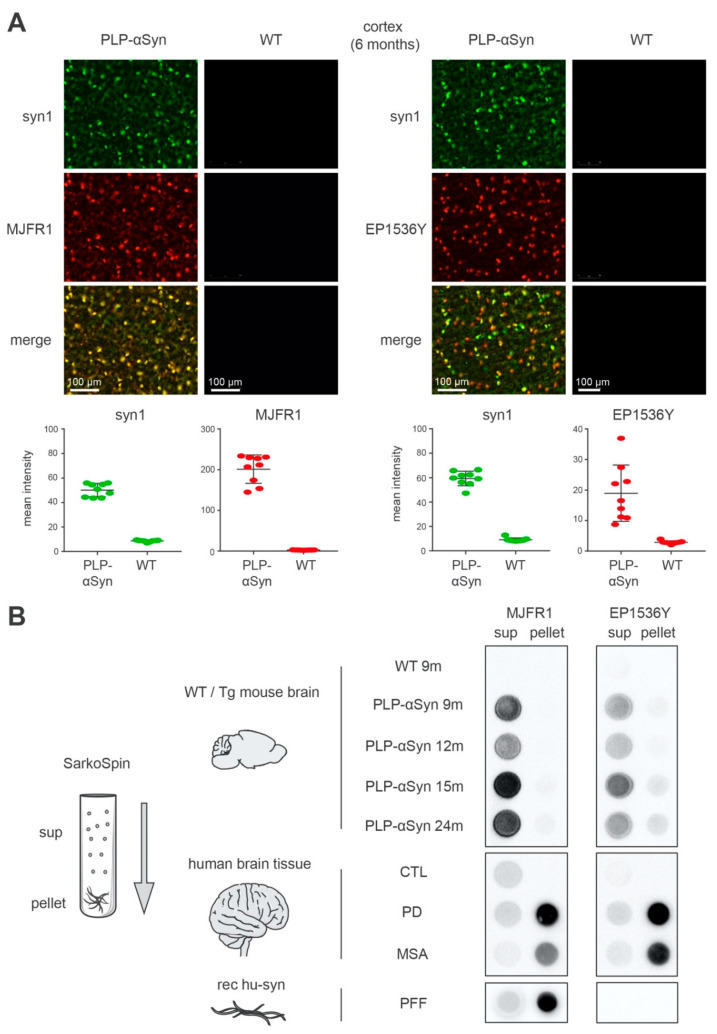
The S129-phosphorylated α-syn species found in the brain of PLP-αSyn mice are distinct from the amyloid forms extracted from Parkinson’s disease (PD) and multiple system atrophy (MSA) brains and from recombinant preformed fibrils (PFFs). (**A**) Immunofluorescence staining of 6 months-old PLP-αSyn mouse cortical sections, representative of 3 independent experiments. Total α-syn (syn1, green, **top panels**) shows a complete colocalization with human α-syn (MJFR1, red, **mid-left panels**) and partial colocalization with pS129 phosphorylated α-syn (EP1536Y, red, **mid-right panels**). The 4 lower panel quantifications depict the mean field intensity values of 9 cortical/striatal fields of views at 20× from the sections shown above and confirm the massive overexpression of α-syn in PLP-αSyn mouse brains compared to wild-type (WT). (**B**) Biochemical aggregation analysis of the different species of α-syn found in WT or PLP-αSyn mouse brains (**top**), control, PD and MSA human subject brains (**middle**) or a preparation of recombinant human α-syn PFF (**bottom**). Pooled 9 to 24 months-old mice (*n = 3*) or human (*n* = 3) brain homogenates and PFF samples were subjected to SarkoSpin procedure consisting of a sarkosyl solubilization at 37 °C with nuclease under shaking followed by an ultracentrifugation on sucrose cushion. The contents in human α-syn (MJFR1, **left panel**) and pS129-α-syn (EP1536Y, **right panel**) of SarkoSpin supernatant and pellet fractions were assessed by filter trap followed by immunolabelling with the respective antibodies. Pictures are representative of *n* = 3 independent Sarkospin procedures quantified in Supplementary Figure S1.

As previously reported using immunohistochemistry [14], double immunofluorescence (Figure 1A) revealed that the OLs populating the brains of PLP-αSyn mice express high levels of human α-syn (Figure 1A, MJFR1 panel). Indicative of a massive overexpression, human α-syn levels in OLs are so high that they account on their own for total brain α-syn levels 500% to 800% higher than in wild-type animals [34]. Thus, image acquisition adapted to detect α-syn in the OLs of PLP-αSyn mice with the species-independent antibody syn1 (Figure 1A, syn1 panels) leaves the endogenous physiological α-syn neuronal signal in the background. Similarly, these acquisition conditions for syn1 also yield a completely dark image for control animals (Figure 1A). In PLP-αSyn mice most of the OLs are also positive for EP1536Y that detects S129-phosphorylated α-syn (Figure 1A, EP1536Y panel). In these conditions brain sections from WT animals are totally negative for EP1536Y (Figure 1A).

The emergence of hyperphosphorylated α-syn in the OLs of PLP-αSyn mice was previously interpreted as the formation of “GCIs” [14,34]. GCIs are cytoplasmic inclusions found in the OLs in the brains of MSA patients that contain fibrillar α-syn [35,36]. We thus proceeded with parallel extractions, sedimentations and filter-trapping of sarkosyl-insoluble amyloid aggregates (see methods) (Figure 1B) from the brains (i) of transgenic animals at different ages (Figure 1B, PLP-αSyn), (ii) of control animals (Figure 1B, WT), (iii) of human control, PD and MSA subjects (Figure 1B, CTL, PD, MSA), as well as from pure recombinant human α-syn preformed fibrils (Figure 1B, PFF).

The results indicate that the method efficiently separated α-syn amyloid fibrils from soluble α-syn forms which coincided with the S129-phosphorylated α-syn pool for PD and MSA brain extracts (see methods) (Figure 1B, PFF, PD, MSA). Indeed, by revealing human α-syn with the MJFR1 antibody, it appeared that over 70% of the total α-syn present in brain extracts from PD and MSA patients was pelleted and trapped, over 90% when synthetic PFFs were used and less than 3% when dealing with brain extracts from control subjects. In PD and MSA patients, S129-phosphorylated α-syn coincided with the insoluble α-syn pool since more than 85% of the phospho-S129 signal (EP1536Y-positive) was found in the pellets. In clear contrast, this figure was less than 3% for the control subjects (Supplementary Figure S1)

In strong contrast to MSA or PD brain extracts however, the same procedure applied to samples from PLP-αSyn mice showed that the fraction of α-syn that could be pelleted and trapped was unexpectedly low, remaining below 7% (Supplementary Figure S1). This indicated that the human α-syn burden that builds up in these animals is not fibrillar like in PD or MSA. Strikingly enough and again in contrast with PD and MSA, the high levels of S129-phosphorylated α-syn present in the brains of the PLP-αSyn mice appeared to correspond to soluble forms as over 80% of the latter were retained in the supernatants while only 3% to 5% was retained in the supernatants for PD and MSA samples (Supplementary Figure S1).

### 3.2. S129-phosphorylated Human α-syn Found in PLP-αSyn Mouse Brains Corresponds to Soluble Monomeric and Oligomeric Forms

**Figure 2:**
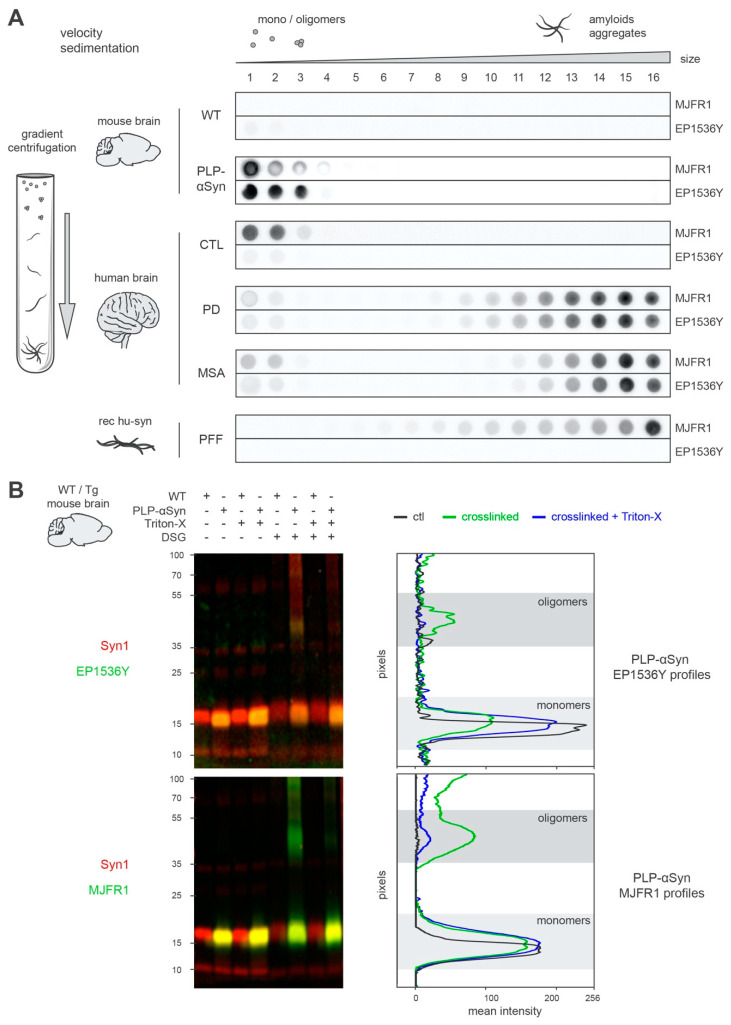
Unlike in MSA and PD, S129-phosphorylated human α-syn is oligomeric and monomeric in PLP-αSyn mouse brains. (**A**) Aggregation profiles obtained by sedimentation of the different species of α-syn found in WT or PLP-αSyn mouse brains (**top**), control, PD and MSA human subject brains (**middle**) or a preparation of recombinant human α-syn PFF (**bottom**). Pooled mouse (*n* = 3) or human (*n* = 3) brain homogenates and PFF samples were subjected to SarkoSpin solubilization followed with fractionation by sedimentation velocity upon ultracentrifugation on iodixanol gradient. The distribution of human α-syn (MJFR1) and pS129-α-syn (EP1536Y) was analyzed by filter trap on the collected fractions (numbered from top to bottom of gradient) followed by immunostaining with the respective antibodies. Pictures are representative of *n* = 3 independent sedimentations quantified in Supplementary Figure S2. (**B**) Representative western blot pictures (**left**) and their intensity quantification (**right**) of WT/PLP-αSyn mouse brains samples. Cytosolic fractions from pooled 9 months-old WT (*n* = 3) or age-matched PLP-αSyn (*n* = 3) brain homogenates were treated or not with Triton-X (0.25%, 30 min on ice) followed by crosslinking or not with DSG (2 mM, room temperature). These samples were subjected to SDS-PAGE electrophoresis and immunoblotted with couples of antibodies directed against total α-syn (syn1, red) and pS129-α-syn (EP1536Y, green, **top**) or human α-syn (MJFR1, green, bottom) using infrared dyes labelled secondary antibodies. Signal intensity of pS129-α-syn (**top**) and human α-syn (**bottom**) were quantified vertically by line scanning for PLP-αSyn samples untreated (black), DSG-crosslinked (green) or with prior Triton-X treatment (blue). Monomeric (light) and oligomeric (dark) species of the two forms of the proteins are depicted with the grey zones.

In order to better characterize the nature of the latter α-syn pool, we proceeded with velocity sedimentations on linear iodixanol gradients that finely separate the α-syn assemblies present in the samples according to size and density (Figure 2A and Supplementary Figure S2). The results confirmed that the phospho S129-positive OL α-syn pool present in PLP-αSyn brains is not fibrillar like in PD or MSA patients. Instead, it sediments at the same levels as the unphosphorylated physiological forms of α-syn found in control human brains (Figure 2A and Supplementary Figure S2), that is, soluble monomers and oligomers. Regarding the pS129-positive α-syn pool present in OLs, SDS-PAGE performed on PLP-αSyn mouse brain extracts only evidenced pS129-positive monomers (Figure 2B). However, prior crosslinking of the extracts with DSG to prevent oligomer disassembly by SDS, allowed the identification of an oligomeric population (Figure 2B), confirming the results obtained in velocity sedimentation experiments (Figure 2A). Interestingly, these oligomers were no longer detected when Triton-X was applied before crosslinking (Figure 2B). With regards to this result, structuration of the oligomers around lipids and/or presence of weak hydrophobic interactions facilitating oligomer assembly can be hypothesized.

Collectively these results indicate that in PLP-αSyn mice (i) fibrillar forms of α-syn are not detectable and (ii) that the pS129-positive OL α-syn pool corresponds to soluble monomers and oligomers and cannot thus be considered as GCIs.

**Figure 3:**
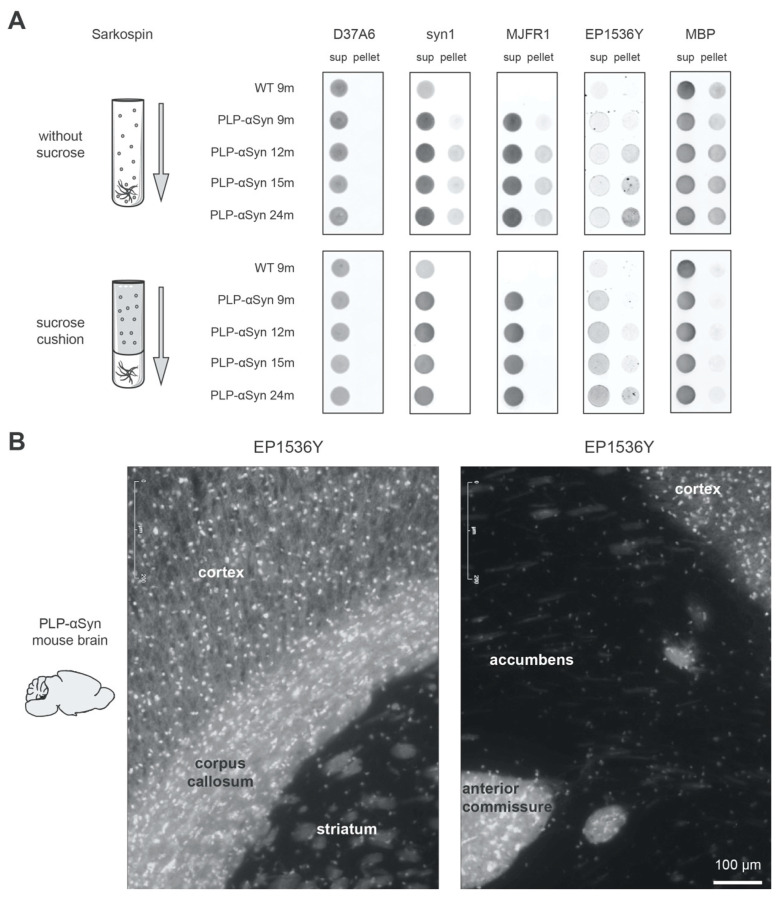
pS129-positive α-syn mono/oligomers co-sediment with MBP and are enriched in myelinated regions of the PLP-αSyn mouse brain. (**A**) Sedimentation analysis of the different species of α-syn found in WT or PLP-αSyn mouse brains with or without myelin floatation. Pooled 9 to 24 months-old mouse brain homogenates (*n* = 3) were subjected to SarkoSpin normal procedure (with sucrose cushion, **bottom**) or to the same procedure without myelin floatation sucrose cushion (without sucrose, **top**). The content in endogenous murine α-syn (D37A6), total α-syn (syn1), human α-syn (MJFR1), pS129-α-syn (EP1536Y) and myelin basic protein (MBP) of SarkoSpin supernatant and pellet fractions were assessed by filter trap followed by immunolabelling with the respective antibodies. Pictures are representative of *n* = 2 independent Sarkospin procedures quantified in Supplementary Figure S3. (**B**) Representative pS129-α-syn immunofluorescence staining of six months-old PLP-αSyn mouse brain sections. Hyperphosphorylated forms of the protein are prominent in regions with long myelinated axons such as the corpus callosum and the anterior commissure.

With regard to their localization in OLs and to the impact of Triton-X, we reasoned that a possibility could be that the pS129-positive monomers and oligomers seen in the transgenic animals could be associated with myelin. To address this hypothesis, we simply ran again the ultracentrifugation separation experiments shown in Figure 1B but removing this time the sucrose cushion meant to float the myelin sheath contaminants present in the brain extracts (Figure 3A). As expected, Figure 3A shows that omission of the sucrose cushion in the separation procedures resulted in a drastic enrichment of the pellet fraction with myelin basic protein (MBP) indicative of myelin sheath sedimentation. The presence of the latter myelin contaminants coincided with the appearance and the reinforcement of a pellet signal (i) for EP1536Y that detects S129-phosphorylated α-syn, (ii) for the anti-synuclein antibodies syn1 and MJFR1 both capable of detecting the human α-syn overexpressed by the OLs, (iii) but not for D37A6 that detects only the endogenous mouse α-syn expressed in neurons. Quantifications of the dot blots shown in Figure 3A are shown in Supplementary Figure 3. These results are compatible with the idea that the pS129-positive monomers and oligomers seen in the transgenic animals can get associated with myelin. They also suggest that previous studies in which no specific care was taken to manage myelin contaminants could have been confounded, the latter “dragging along” soluble monomer and oligomers of α-syn into the pellet.

Interestingly, the notion of association with myelin is also supported by the immunofluorescence mapping of the pS129-positive α-syn pool in brain sections of the transgenic animals (Figure 3B). Indeed, a very strong and diffuse increase of EP1536Y signal is observable in regions rich in myelinated axons like the corpus callosum and the anterior commissure (Figure 3B).

### 3.3. Is the Presence of Neuronal Human α-syn in PLP-αSyn Primary Cortical Cultures due to an OL-to-Neuron Transfer of the Protein?

In spite of the absence of fibrillar amyloid forms of α-syn in their brains, the PLP-αSyn mice yet exhibit a partial neuronal depletion in the substantia nigra and the striatum (reviewed in Reference [24]) as well as lesions in brainstem nuclei such as the pre-Bötzinger complex [24] and have been reported to develop defects in the execution of motor tasks as well as autonomic dysfunctions [25,28,29,34,37].

**Figure 4:**
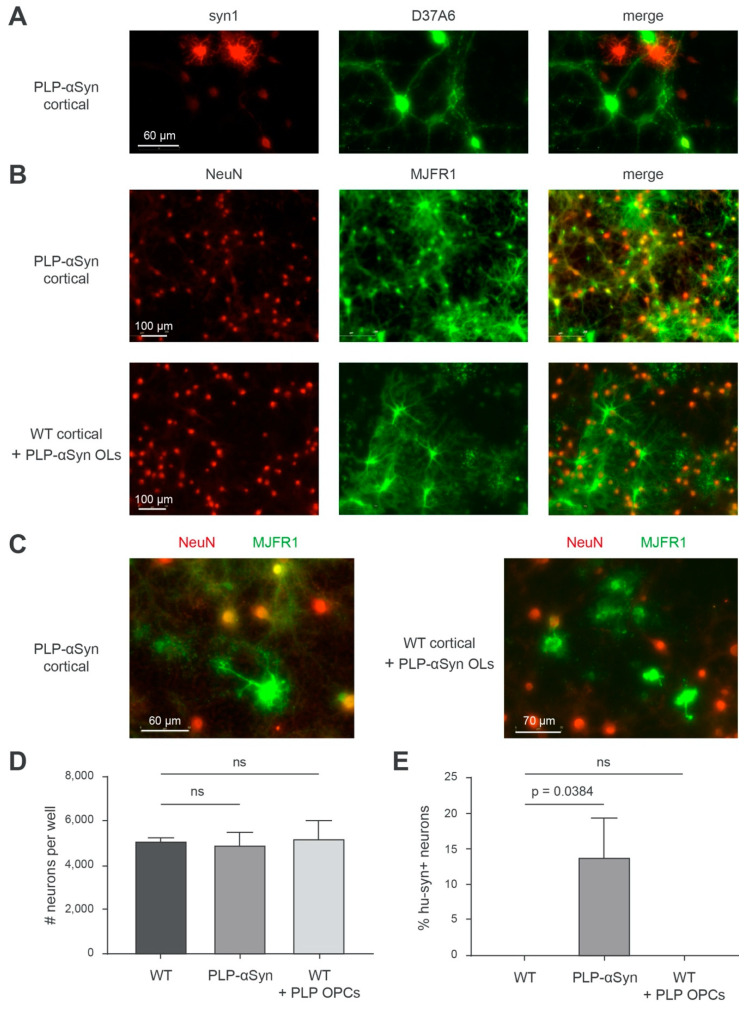
The presence of neuronal human α-syn in PLP-αSyn primary cortical cultures is not due to an OL-to-neuron transfer of the protein. (**A**) Representative immunofluorescence imaging of total α-syn (syn1, red) and endogenous murine α-syn (D37A6, green) in primary cortical cultures PLP-αSyn mouse. (**B**) Representative immunofluorescence imaging of neurons (NeuN, red) and human α-syn (MJFR1, green) in primary cortical cultures from PLP-αSyn mouse (**top**) or WT mouse supplemented with PLP-αSyn oligodendrocytes (**bottom**). (**C**) Close-up representative illustrations of the merged pictures shown in B. (**D**) Bar graph of the quantifications of the number of neurons (NeuN+ cells) per well. Equal averages of approximately 5000 neurons were obtained, with no significant differences between conditions (Holm-Sidak corrected multiple *t*-tests), ns: not statistically different. (**E**) Bar graph of the quantifications of the ratios of human α-syn positive neurons (hu-syn+ NeuN+/NeuN+ cells) per well in the three different culture conditions. After thresholding out the residual unspecific nuclear staining yielded by MJFR1, these cells are detected solely in PLP-αSyn primary cortical cultures (*p*-Values obtained with Holm-Sidak corrected multiple *t*-tests). For each condition 9 fields corresponding to 5.13 mm^2^ that is, 15% of the total well surface, of 2 independent wells were analyzed. Results are representative of 3 independent experiments.

One possibility we thus considered was that a fraction of the α-syn produced by the OLs could be transferred to neurons and could disturb their normal function. To understand if such a crosstalk between OLs and neurons could take place in these mice, we studied the α-syn pools in primary cortical cultures containing OLs and neurons both derived from PLP-αSyn mice and compared it with primary cortical cultures from WT animals enriched with OLs isolated from PLP-αSyn mice (Figure 4). If a transfer of α-syn from the OLs to the neurons was present, the protein should be observable in both types of cultures. Figure 4A shows that the OLs express high levels of human α-syn (strongly syn1 positive, D37A6 negative) in DIV 30 fully differentiated primary cortical cultures from PLP-αSyn mice and that neurons endogenously express mouse α-syn (barely syn1-positive with these imaging settings, D37A6 positive). Concomitant imaging of neurons with NeuN and of human α-syn with MJFR1 in these cultures (Figure 4B) (and thresholding out the low unspecific nuclear signal yielded by MJFR1) indicated, however that a significant number of neurons (~15%) also contain human α-syn (Figure 4B upper panel row, Figure 4C left panel, Figure 4E) compatible with the hypothesis of a transfer of human α-syn from OLs to the neurons. However, DIV 30 cortical cultures from WT mice enriched with OLs from PLP-αSyn mice did not show signs of such a transfer, in spite of a strong expression of human α-syn by the OLs from the transgenic animals (Figure 4B, lower panel row, Figure 4C right panel, Figure 4E). This indicates that the presence of human α-syn in the neurons of PLP-αSyn mice is probably not due to a transfer from the OLs but rather to an endogenous expression leak of the PLP promoter in the neurons. Note that it could be argued that the human α-syn found in neurons in the cultures from PLP-αSyn mice derives from a transfer having taken place beforehand, that is, during embryonic life, before the culture phase. This however seems unlikely because it takes several weeks in culture before the primary OLs start expressing human α-syn (Supplementary Figure S4A) suggesting that the PLP promoter of the transgene is not significantly activated in OLs during embryonic life. It thus becomes conceivable that the neuronal pathology and dysfunction seen in the PLP-αSyn mice could be attributable, at least in part, to a direct neuronal effect due to a mis-targeted expression of human α-syn in neurons. Note that in our conditions, the number of live neurons (NeuN positive) in the DIV 30 primary cortical cultures was similar for WT and PLP-αSyn mice and was not modified in the WT cultures enriched with OLs from PLP-αSyn mice (Figure 4D).

### 3.4. S129-Phosphorylated Human α-Syn Is Enriched at “Hot Spots” in OL Processes

**Figure 5:**
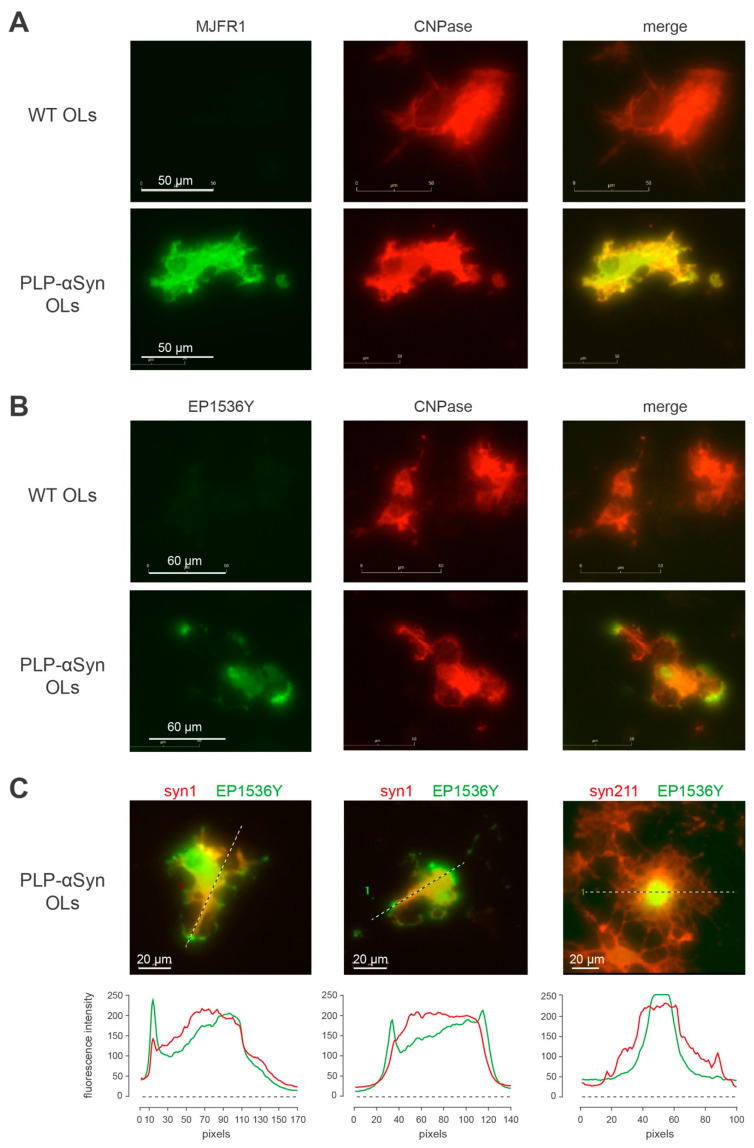
pS129 positive human α-syn is enriched in OL processes (**A**) Representative immunofluorescence imaging of 2′,3′-cyclic-nucleotide 3′-phosphodiesterase (CNPase, red) together with human α-syn (MJFR1, green) in primary cultures of OLs from PLP-αSyn mice and wt mice. (**B**) Representative immunofluorescence imaging of 2′,3′-cyclic-nucleotide 3′-phosphodiesterase (CNPase, red) together with pS129-α-syn (EP1536Y, green, bottom) in primary cultures of OLs from PLP-αSyn mice and wt mice. (**C**) Left and middle panels: localization at the processes tips of phosphorylated human α-syn, with total α-syn (syn1, red) and pS129-α-syn (EP1536Y, green) immunofluorescence staining and respective quantification of the profile intensities by linescan analysis. Right panel shows the occasional nuclear localization of phosphorylated human α-syn, with total human α-syn (Syn-211, red) and pS129-α-syn (EP1536Y, green) immunofluorescence staining and respective quantification of the profile intensities by linescan analysis. Results are representative of 3 independent experiments.

In order to better understand how OLs from PLP-αSyn mice manage to accommodate exceptionally high levels of human α-syn [34] without experiencing α-syn fibrillization, we imaged the subcellular distribution of pS129-positive (i.e., monomers and oligomers) as well as the unphosphorylated α-syn pools in purified OL cultures and cortical cultures from transgenic animals (Figure 5 and Supplementary Figure S4B). At DIV20, purified OLs express the differentiation marker CNPase (Figure 5A CNPase) with human α-syn showing a corresponding diffuse cytoplasmic staining pattern (Figure 5A, MJFR1, merge) instead, and, reminiscent of what is also observed in melanoma cells [38], pS129-positive α-syn exhibits a specific compartmentalized distribution pattern with “hot spots” delineating the tip of certain cellular processes as well as lamellipod-like plasma membrane regions (Figure 5B–C, EP1536Y, merge) [39]. This is particularly evident in OLs in which unphosphorylated and phosphorylated α-syn forms were concomitantly revealed (Figure 5C, left and middle panels), with the additional observation of a variable association of pS129-positive α-syn with the nucleus (Figure 5C, right panel).

Collectively, these results show that second to overexpression, fibrillization of α-syn does not take place in PLP-αSyn mice and that the phosphorylated fraction of α-syn remains monomeric and oligomeric and gets associated with the nucleus and with specialized regions of the cellular membrane [39]. This indicates that differentiated OLs that turned off their endogenous α-syn expression [6], can accommodate the experimental overexpression of human α-syn, phosphorylate monomers and oligomers and direct the latter in specific sub-cellular compartments. It is possible that our observations regarding PLP-αSyn mice could also hold true for CNP- and MBP-αSyn mice [15,16] and, more generally, for other targeted α-syn overexpression strategies. The fact that the fibrillization process of α-syn does not take place in the OLs of PLP-αSyn mice despite very high α-syn expression levels is particularly intriguing and should help in the identification of the signaling pathways capable of inducing α-syn amyloid aggregation.

### 3.5. Aggregation of Human α-Syn Does Not Spontaneously Take Place on OLs from PLP-αSyn Mice but Can Be Experimentally Seeded with PFFs

**Figure 6:**
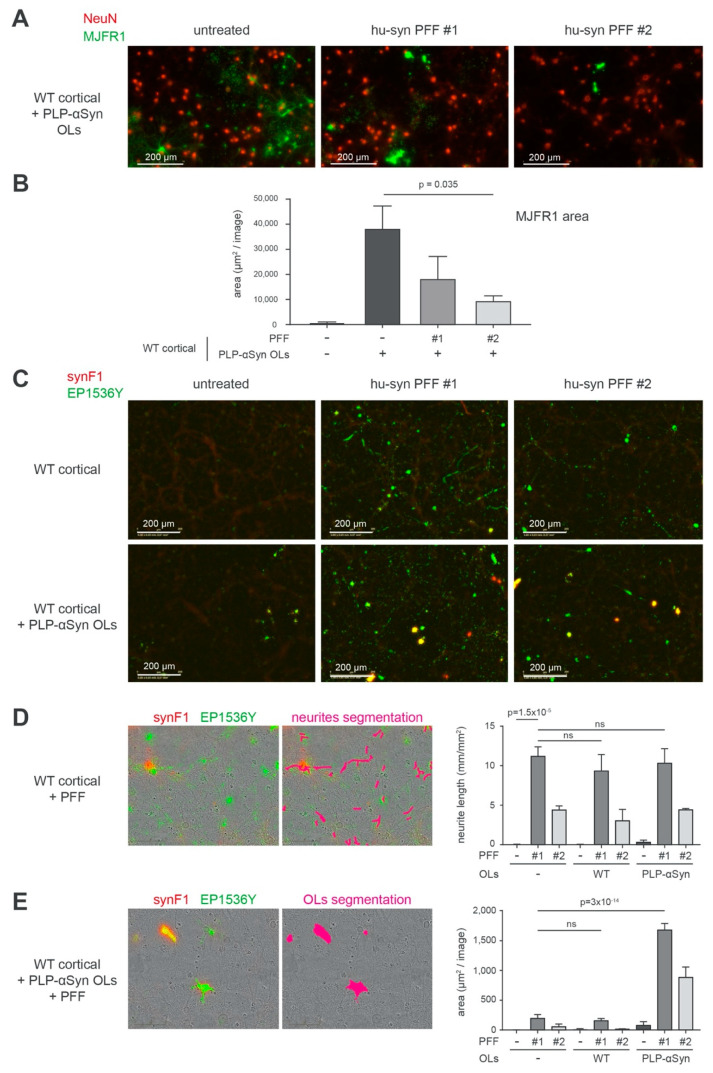
Aggregation of human α-syn does not spontaneously take place on OLs from PLP-αSyn mice but can be experimentally seeded by challenging with PFFs. (**A**) Representative immunofluorescence imaging of neurons (NeuN, red) and human α-syn (MJFR1, green) in primary cortical cultures from WT mouse supplemented with OLs from PLP-αSyn mice, untreated (**left**) or challenged with human α-syn PFF of type 1 (**middle**, PFF #1) or type 2 (right, PFF #2). Pictures were taken four weeks post treatment and show OLs morphology changes with cellular shrinking upon PFF treatments. (**B**) Bar graph representing the quantification of MJFR1 area in the different conditions described in A, traducing the OLs surface measurements, with a significant shrinking of these cells upon challenge with PFF (pValues obtained with Holm-Sidak corrected multiple *t*-tests). (**C**) Representative immunofluorescence imaging of the synucleinopathy as shown by aggregated α-syn (synF1, red) and hyperphosphorylated pS129-α-syn (EP1536Y, green) in primary cortical cultures from WT mouse (**top**) and the same cultures supplemented with OLs from PLP-αSyn mice (**bottom**). These two cultures were untreated (**left**) or challenged with human α-syn PFF of type 1 (**middle**, PFF #1) or type 2 (**right**, PFF #2). (**D**) Representative image shown in C with a neurite segmentation filter applied (right, pink), allowing the quantification of neuritic synucleinopathy plotted in the bar graph. Total synucleinopathy neurite length is shown for WT primary cortical cultures supplemented or not with WT or PLP-αSyn OLs and challenged or not with PFF of the two types. The extent of the neuronal synucleinopathy is independent of the addition of OLs (*p* values obtained with Holm-Sidak corrected multiple *t*-tests), ns: not statistically different. (**E**) Representative image shown in C with an OLs segmentation filter applied (**right**, pink), allowing the quantification of synucleinopathy located in the OL cell bodies, plotted in the bar graph. Total OLs synucleinopathy is shown as area measured for WT primary cortical cultures supplemented or not with WT or PLP-αSyn OLs and challenged or not with PFF of the two types (*p* values obtained with Holm-Sidak corrected multiple *t*-tests). For each condition 9 fields of 2 independent wells were analyzed corresponding to 5.13 mm^2^ that is, 15% of the total well surface. Results are representative of 2 independent experiments.

Interestingly, we found that α-syn amyloid aggregation could be induced in OLs by seeding the cortical cultures from PLP-αSyn mice with exogenous preformed fibrils made of human α-syn (PFFs) (Figure 6) [30]. In Figure 6A, B we challenged the cultures with 2 different PFF production batches [30] (see also methods section) and observed a drastic morphological change of the MJFR1 signal distribution in OLs corresponding to a “contraction” compatible with an aggregation of α-syn induced by the exogenous PFFs in these cells. As expected, PFF treatment induced a canonical pS129-positive neuronal synucleinopathy at the expense of endogenous α-syn in WT cultures (Figure 6C, upper row panel and Supplementary Figure S5). In cultures enriched with OLs from PLP-αSyn mice (Figure 6C, lower row panel), PFFs induced a massive increase of the EP1536Y signal that coincided with a strong positivity with the aggregate-specific antibody synF1 [40]. This indicated that PFFs were able to seed the fibrillization process in OLs overexpressing α-syn. Note that the neuronal aggregates seen in the upper panel row of Figure 6C are also synF1 positive but that the signal amplification chosen to document the process in OLs left this signal close to the background in the images shown. Using the topology of the EP1536Y signal, we performed a differential quantification of the fibrillization process induced by PFFs in neurons (Figure 6D) and in the OLs (Figure 6E) that confirmed this observation. For quantifying pS129-positive α-syn in neurons, the dedicated Incucyte image analysis module allowing the specific segmentation of neuritic fluorescence signals was used. For quantifying aggregated α-syn in OLs a double segmentation of cell bodies based on synF1 basal signal linked to overexpression and on EP1536Y was used.

The capability of PFFs to induce fibrillization in the OLs indicates that overexpression of α-syn alone is not sufficient to trigger α-syn nucleation but that the α-syn pool derived from overexpression is available for templating by exogenous seeds.

In conclusion, while PLP-αSyn mice do not spontaneously develop a fibrillar synucleinopathy similar to MSA patients, their OLs can however be induced to do so by using exogenous PFFs as seeds. Interestingly, early imaging of the primary PLP-αSyn cultures only 4 days after seeding with the PFFs revealed that they decorated the surface of the neurons but not that of the OLs (Supplementary Figure S6) revealing possible different PFF uptake modes in the two cell types.

In agreement with a recent report concerning wild-type mice [41], intracerebral injections of PFFs in PLP-αSyn mice could thus represent a way to model a *bona fide* fibrillar synucleinopathy in OLs in vivo. Besides the development of such a model, next steps will also consist in understanding why α-syn overexpression in the OLs of the PLP-αSyn mice results in the accumulation of phosphorylated monomers and oligomers instead of yielding a pathological burden of phosphorylated amyloid fibrils.

### 3.6. Considerations about the Face vs. Predictive Value of the PLP-αSyn Mouse Model

The new data presented here show that the α-syn OL load present in the brains of PLP-αSyn mice cannot be compared to the GCIs that characterize MSA. The former is constituted of large amounts of phosphorylated monomers and oligomers that are neither observed in MSA or PD patients nor in control subjects (Figure 2 and Supplementary Figure S2), while the latter are filled with typical α-syn amyloid fibrillar species that very well compare with the ones found in PD patients and with pure synthetic α-syn fibrils (Figure 2 and Supplementary Figure S2).

It should be highlighted that our observations do not support the idea that GCIs or Lewy Bodies do not contain α-syn amyloids [42]: instead, our analysis reveals the prominent presence of amyloid fibrils, which is in agreement with numerous previous studies [35,36] but importantly, it also points to the absence of phosphorylated monomers and oligomers in the extracts of MSA and PD patients that could differentiate them from control subjects (Figure 2 and Supplementary Figure S2).

The absence of fibrillar α-syn amyloid species in the brain of the PLP-αSyn mice seems to be in line with the notion put forward by certain authors that the clinical manifestations seen in these animals are “mild” [24,25] compared to MSA. In particular, while MSA is rapidly evolutive and causes the premature death of the patients, the lifespan of PLP-αSyn mice is normal [24]. This later observation is striking if one considers that their total brain α-syn levels are 500 to 800% higher than in wild-type animals [34]. It thus seems a reasonable hypothesis to consider that the lack of formation of amyloid species in these transgenic mice can be responsible for the mildness of the clinical signs they exhibit.

According to our results and irrespective of their intensity, the clinical signs that develop in PLP-αSyn mice could thus have two non-mutually exclusive origins: (i) the existence of an expression leak in neurons that could cause direct detrimental effects in these cells and (ii) the steady presence of a high load of phosphorylated monomers and oligomers in the OLs leading to their progressive dysfunction. Whether this latter possibility could correspond to a mechanism mimicking a process taking place in MSA is not supported by our present data which indicate the absence of accumulation of phosphorylated monomers and oligomers in the extracts of MSA and PD patients compared to control subjects. It could be hypothesized that in the early stages of MSA, a transient peak of phosphorylated monomeric and oligomeric α-syn species could precede and give way to the appearance of GCIs [13]. Whether clinical signs of MSA can be ascribed to the early and transient presence of such assemblies remains, however, to be explored. Thus, in spite of a documented face value of the PLP-αSyn mouse animal model, it appears premature to conclude on its predictive value.

## Figures and Tables

**Table 1:** List of antibodies used in the study.

Antibody	Target	Company	Cat.No	Dilution IF	Dilution IB
**Primary antibodies**					
MJFR-1	human alpha-synuclein	Abcam	ab138501	1: 1000	1: 10,000
EP1536Y	pS129 phospho-synuclein	Abcam	ab51253	1: 500	1: 5000
Syn1 clone 42	human and murine alpha-synuclein	BD Biosciences	610787	1: 500	1: 2000
D37A6	murine alpha-synuclein	Cell Signaling	#4179	1: 200	1: 2000
SynF1	aggregated alpha-synuclein	BioLegend	847802	1: 500	1: 10,000
syn211	human alpha-synuclein	Abcam	ab80627	1: 500	n/a
MBP	myelin basic protein	Abcam	ab218011	1: 200	1: 2000
CNPase	cyclic nucleotide phospho.	Abcam	ab6319	1: 500	n/a
Sox10	sox10 protein	Abcam	ab155279	1: 200	n/a
NeuN	neuronal nuclei protein	Merck Millipore	MAB377	1: 500	n/a
Actin	beta-actin	Sigma	A5316	n/a	1: 10,000
**Secondary antibodies**					
Goat anti-mouse HRP	mouse IgG (H + L)	Jackson Immuno	115-035-146	n/a	1: 10,000
Goat anti-rabbit HRP	rabbit IgG (H + L)	Jackson Immuno	111-035-144	n/a	1: 10,000
Goat anti-mouse IRDye 680RD	mouse IgG (H + L)	LI-COR	926-68070	n/a	1: 5000
Goat anti-rabbit IRDye 800CW	rabbit IgG (H + L)	LI-COR	926-32211	n/a	1: 5000
Donkey anti-mouse Alexa 488	mouse IgG (H + L)	Thermo Fisher Sci.	A-21202	1: 500	n/a
Goat anti-rabbit Alexa 488	rabbit IgG (H + L)	Thermo Fisher Sci.	A-11008	1: 500	n/a
Donkey anti-mouse Alexa 594	mouse IgG (H + L)	Thermo Fisher Sci.	A-21203	1: 500	n/a
Donkey anti-rabbit Alexa 594	rabbit IgG (H + L)	Thermo Fisher Sci.	A-21207	1: 500	n/a

IF: Immunofluorescence; IB: Immunoblotting.

## Supplementary Materials

The following are available online at https://www.mdpi.com/2073-4409/9/11/2371/s1, Figure S1: Quantification of α-syn species found in the different Sarkospin fractions, Figure S2: Human α-syn and pS129-α-syn sedimentation profiles in mouse or human brain and recombinant PFF samples, Figure S3: Quantification of different α-syn species and MBP found in Sarkospin fractions, Figure S4: Human α-syn is highly expressed in primary cultures of OLs from PLP-αSyn mice, Figure S5: Neuronal induced synucleinopathy upon challenging primary cortical cultures with human α-syn PFF, Figure S6: Specific neuronal association of fibrillar α-syn upon challenging primary cortical cultures with human α-syn PFFs, Table S1: List of chemicals used the study.
