# Supplementary material for: Overexpression of α-Synuclein by Oligodendrocytes in Transgenic Mice Does Not Recapitulate the Fibrillar Aggregation Seen in Multiple System Atrophy
Source: Cells. 2020 Oct 29;9(11):2371. doi: 10.3390/cells9112371 (PMC7693764; doi:10.3390/cells9112371)
Supplement: Supplementary file 1 [file cells-09-02371-s001.pdf]

Supplementary Material

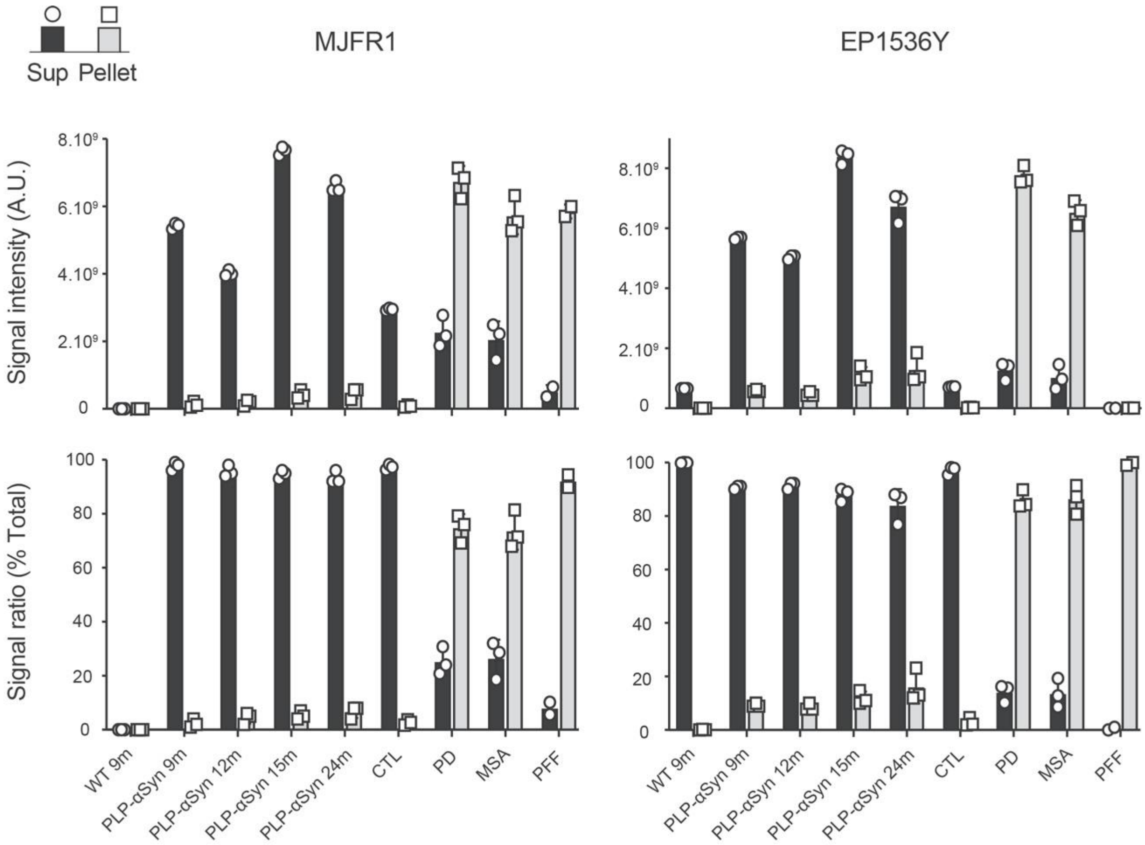

Supplementary Figure S1. Quantification of α-syn species found in the different Sarkospin fractions. Quantifications of n = 3 independent biochemical aggregation analysis illustrated in Figure 1 of the different species of α-syn found in WT or PLP-αSyn mouse brains, control, PD and MSA human subject brains or a preparation of recombinant human α-syn PFF. Pooled 9 to 24 months-old mice (n = 3) or human (n = 3) brain homogenates and PFF samples were subjected to SarkoSpin procedure consisting of a sarkosyl solubilization at 37 °C with nuclease under shaking followed by an ultracentrifugation on sucrose cushion. The contents in human α-syn (MJFR1, left) and pS129-α-syn (EP1536Y, right) of SarkoSpin supernatant and pellet fractions were assessed by filter trap followed by immunolabelling with the respective antibodies. Total intensity (top, A.U.) and signal ratio (bottom, % of total) are plotted for supernatant (dark grey, circles) and pellet (light grey, squares) fractions of each sample and antibody for n = 3 independent Sarkospin procedures (mean +/− SD and individual values).

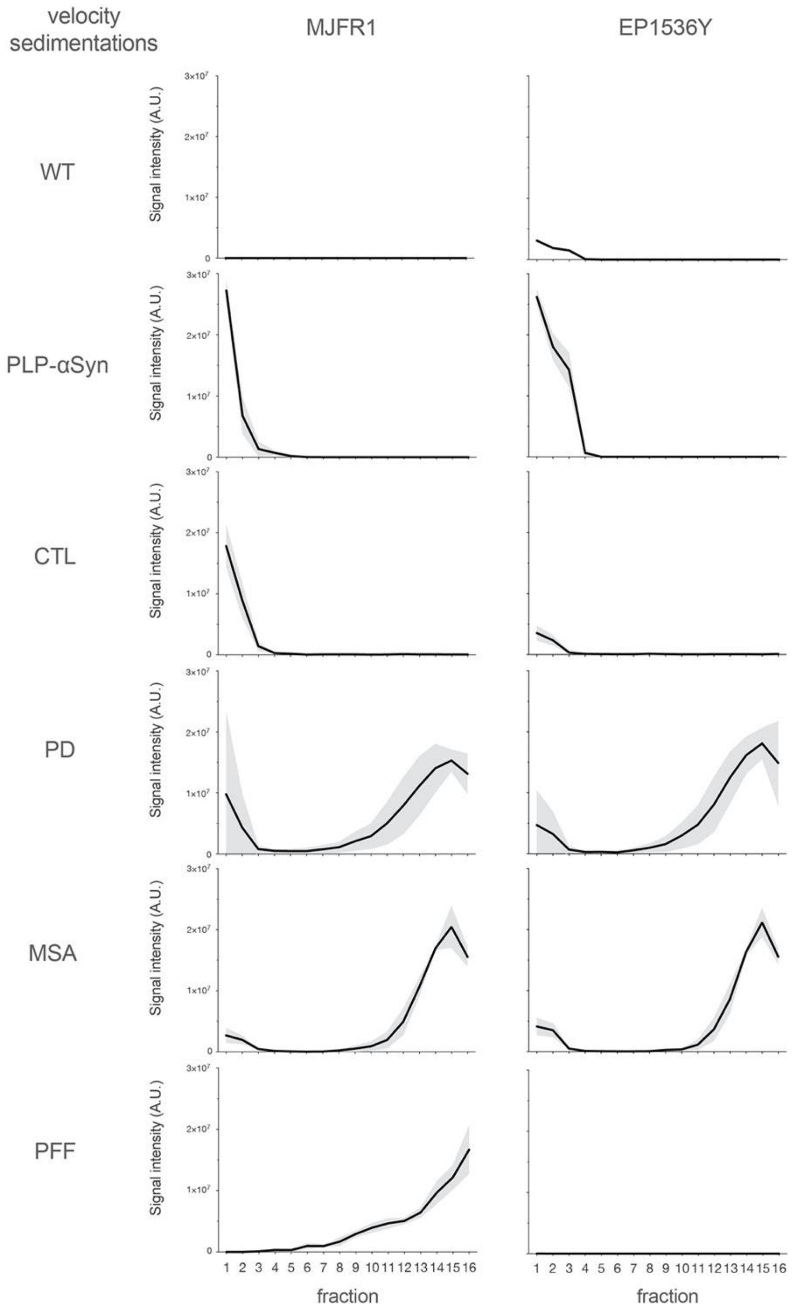

Supplementary Figure S2. Human α-syn and pS129-α-syn sedimentation profiles in mouse or human brain and recombinant PFF samples. Aggregation profiles obtained by n = 3 independent velocity sedimentations of WT or PLP-αSyn mouse brains (top), control, PD and MSA human subject brains (middle) or a preparation of recombinant human α-syn PFF (bottom). Pooled mouse (n = 3) or human (n = 3) brain homogenates and PFF samples were subjected to SarkoSpin solubilization followed with fractionation by sedimentation velocity upon ultracentrifugation on iodixanol gradient. The distribution of human α-syn (MJFR1, left) and pS129-α-syn (EP1536Y, right) was analyzed by filter trap on the collected fractions (numbered from top to bottom of gradient) followed by immunostaining with the respective antibodies. Signal intensity (A.U.) of each fraction was plotted for all samples and antibodies as a bold line (mean) and shaded area (+/− SD).

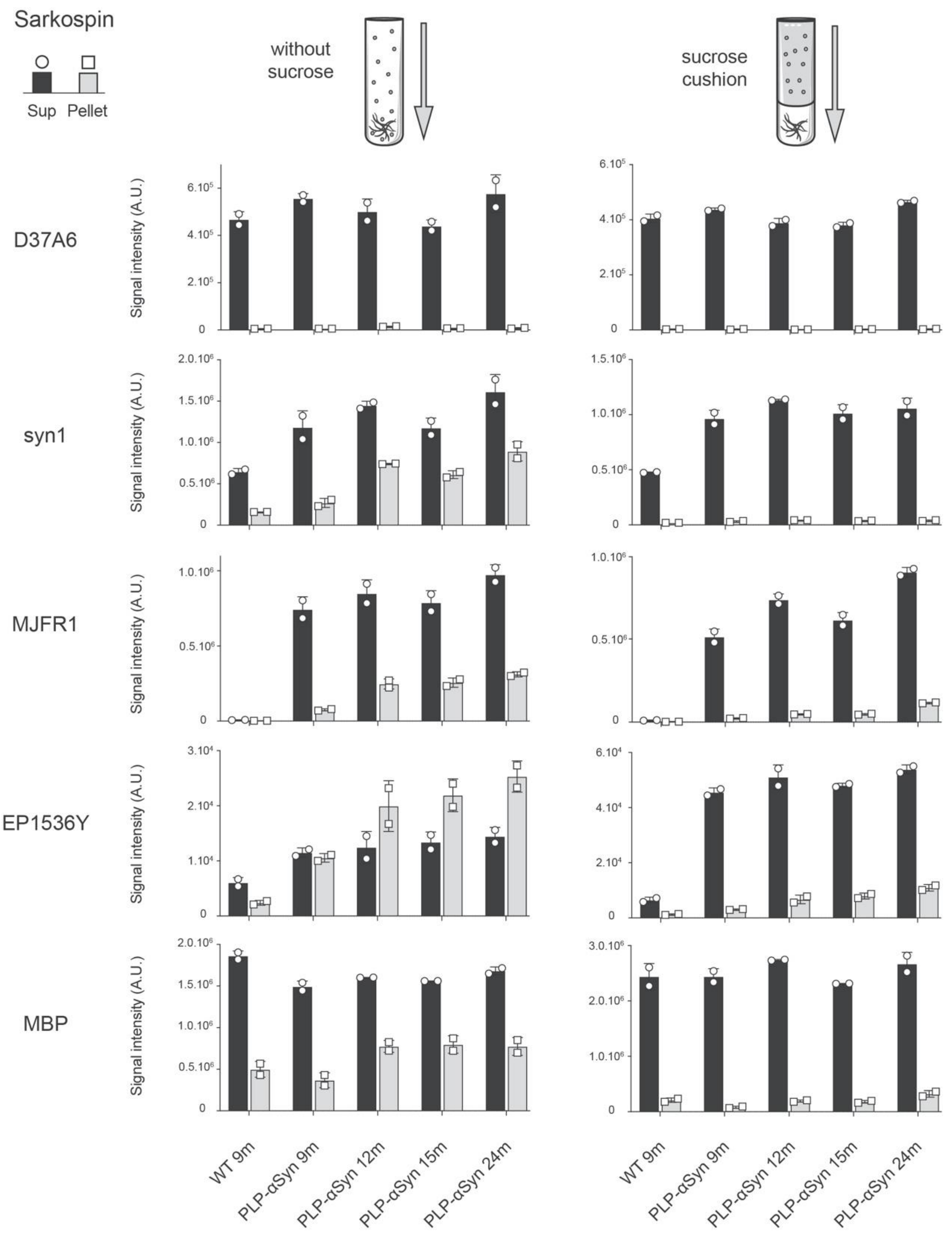

Supplementary Figure S3. Quantification of different α-syn species and MBP found in Sarkospin fractions. Quantifications of n = 2 independent biochemical aggregation analysis illustrated in Figure 3 of the different species of α-syn found in WT or PLP-αSyn mouse brains. Pooled 9 to 24 months-old mice (n = 3) brain homogenates were subjected to SarkoSpin procedure consisting of a sarkosyl solubilization at 37 °C with nuclease under shaking followed by an ultracentrifugation without (left) or with (right) sucrose cushion. The contents in rodent α-syn (D37A6), total α-syn (syn1), human α-syn (MJFR1), pS129-α-syn (EP1536Y) and myelin basic protein (MBP) of SarkoSpin supernatant and pellet fractions were assessed by filter trap followed by immunolabelling with the respective antibodies. Total intensity (A.U.) is plotted for supernatant (dark grey, circles) and pellet (light grey, squares) fractions of each sample and antibody for n = 2 independent Sarkospin procedures (mean +/− SD and individual values).

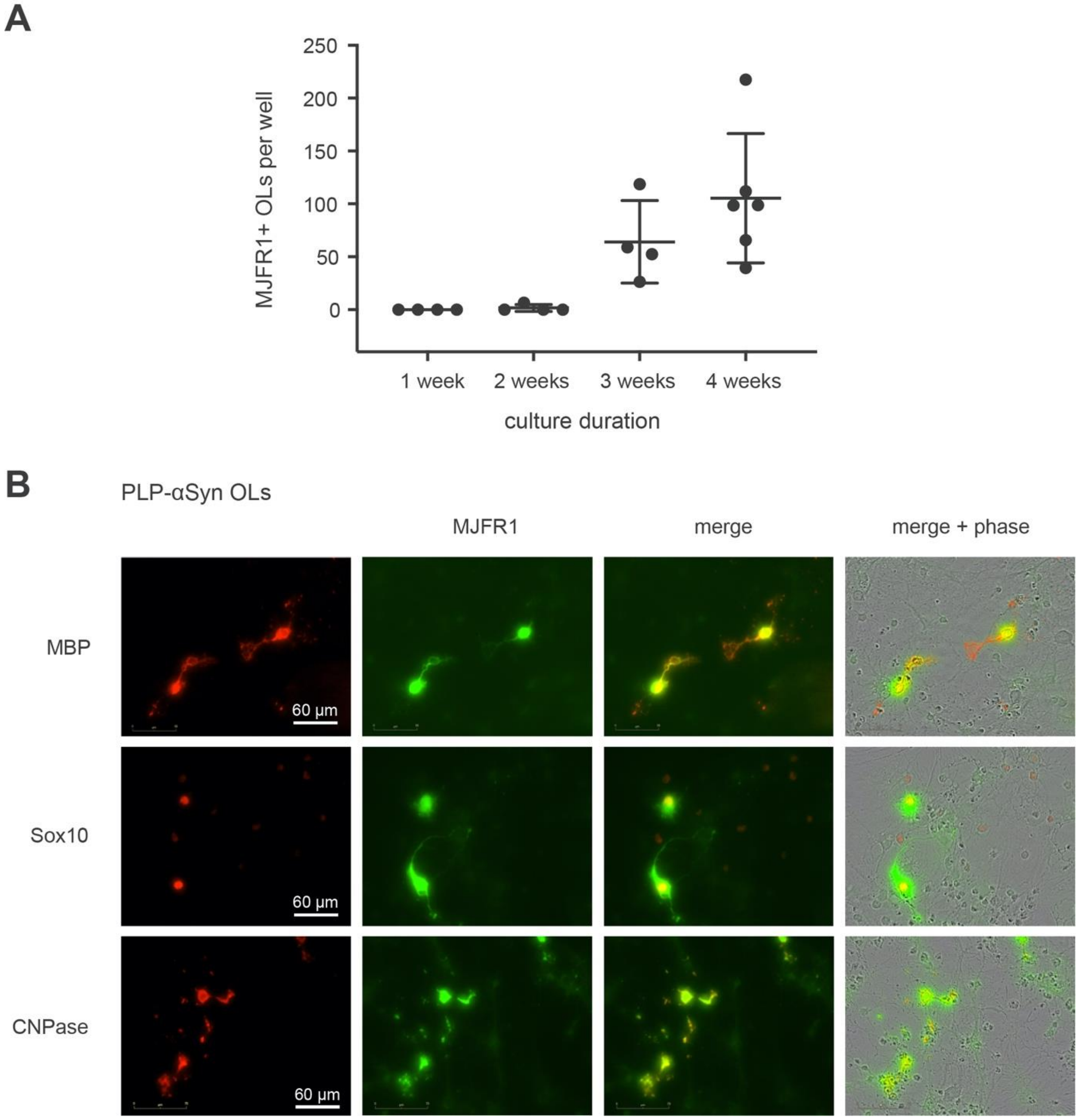

Supplementary Figure S4. Human α-syn is highly expressed in primary cultures of OLs from PLP-αSyn mice. (**A**) Quantifications of the number of MJFR1 positive OLs (i.e., number of cell bodies positive for both CNPase and MJFR1) per well in PLP-αSyn primary cortical cultures at 1, 2 or 3 weeks *in vitro*. Each point corresponds to a single well (96 well plate format). Cells were counted in 9 fields of view of each well, in the three different cultures. The total number of OLs per well was extrapolated by multiplying the value of the 9 fields by the well/field surface ratio. (**B**) Representative immunofluorescence imaging of myelin basic protein (MBP, red, top), transcription factor Sox10 (Sox10, red, middle) and 2′,3′-cyclic-nucleotide 3′-phosphodiesterase (CNPase, red, bottom) together with human α-syn (MJFR1, green) in primary cultures of OLs from PLP-αSyn mice. Human α-syn is specifically highly expressed in MBP+ Sox10+ CNPase+ cells in these cultures. Results are representative of 3 independent experiments

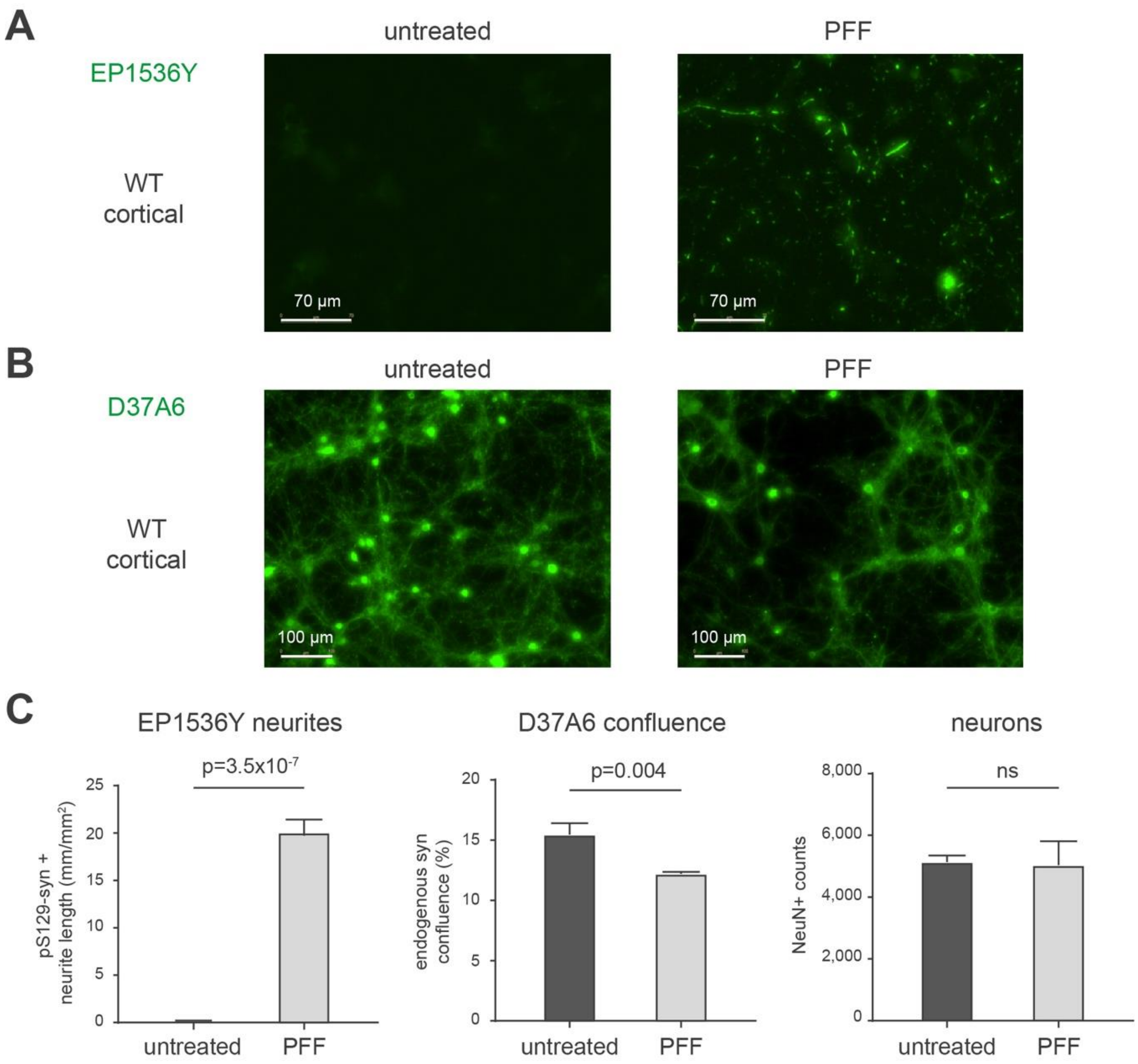

Supplementary Figure S5. Neuronal induced synucleinopathy upon challenging primary cortical cultures with human α-syn PFF. (**A**) Representative immunofluorescence imaging of hyperphosphorylated pS129-α-syn (EP1536Y, green) in primary cortical cultures from WT mice, untreated (left) or challenged with human α-syn PFFs #1 (right). Pictures were taken four weeks post treatment and show neuritic synucleinopathy induced by PFF treatment allowing its quantification represented in the bar graph in C. (**B**) Representative immunofluorescence imaging of endogenous murine α-syn (D37A6, green) in primary cortical cultures from WT mice, untreated (left) or challenged with human α-syn PFF (right). Pictures were taken four weeks post treatment. (**C**) Total pS129-α-syn+ neurite length (left), endogenous α-syn confluency (middle) and neurons (NeuN+ cells, right) are plotted for WT primary cortical cultures challenged or not with PFF (p values obtained with Holm-Sidak corrected multiple *t*-tests), ns: not statistically different. For the total neuritic length presenting a pS129 α-syn signal the EP1536Y signal was quantified using the Incucyte module dedicated to neurite quantification. Note that the induction of neuritic synucleinopathy by PFFs takes place in a limited number of neurons as witnessed by the much more widespread D37A6 signal, detecting total endogenous mouse α-syn, which in this case was expressed as percentage of confluence in the well. The Neurons were counted by segmenting NeuN-positive cell bodies (images not shown). For each condition 9 fields of 2 independent wells were analyzed corresponding to 5,13 mm², that is, 15% of the total well surface. Results are representative of 2 independent experiments

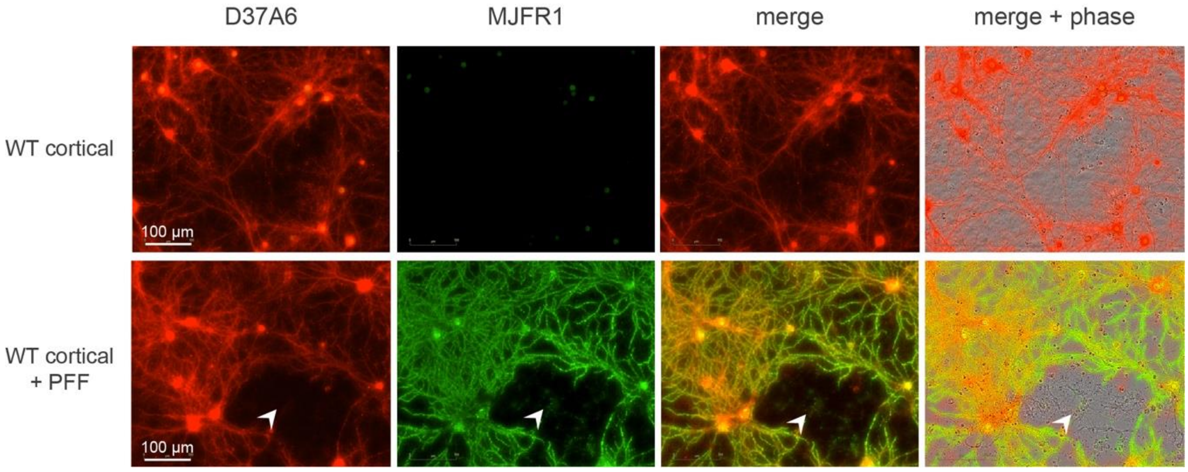

Supplementary Figure S6. Specific neuronal association of fibrillar α-syn upon challenging primary cortical cultures with human α-syn PFFs. Representative immunofluorescence imaging of endogenous murine α-syn (D37A6, red), human α-syn (MJFR1, green) in primary cortical cultures from WT mice, untreated (top) or challenged with human α-syn PFFs (bottom) at 4 DPE. Noteworthy, while neurons show a massive association of human fibrillar α-syn, OLs are totally devoid of the protein (arrowheads pointing at an OL cluster showing almost no human and murine α-syn, while morphologically identified on phase contrast).

Supplementary Table S1. List of chemicals used the study

Name	Company	Cat.No
**Chemicals**		
Neuronal Macs medium	Miltenyi Biotech	130-093-570
Neurobrew-21 supplement	Miltenyi Biotech	130-093-566
FGF-2	Miltenyi Biotech	130-093-839
PDGF-A	Miltenyi Biotech	130-108-983
CTNF	Miltenyi Biotech	130-108-972
Brainphys	Stemcell Technologies	#05790
Complete EDTA-free protease inhibitors	Roche	11873580001
PhosSTOP phosphatase inhibitors	Roche	4906845001
N-lauroyl-sarcosine (sarkosyl)	Sigma	61743
Benzonase nuclease	Novagen	70746-4
Optiprep (iodixanol 60% w/v)	Sigma	D1556-250ML
PFA (37% v/v in MetOH 10% v/v)	Sigma	252549
Triton X-100	Sigma	T8787-100ML
Tween-20	Sigma	P9416-100ML
Bovine Serum Albumin	Sigma	810533
Disuccinimidyl Glutarate (DSG)	Sigma	80424-50MG-F
**Kits**		
Neuronal tissue dissociation kit (C-Tubes)	Miltenyi Biotech	130-095-937
CD140a (PDGFRα) MicroBead Kit (C-Tubes)	Miltenyi Biotech	130-093-237
Tissue homogenization kit (M-Tubes)	Miltenyi Biotech	130-093-236
SDS-PAGE Protean TGX Gel migration	Biorad	4561045
SDS-PAGE Transfer Transblot Turbo	Biorad	1704159
